# Household transmission of SARS-CoV-2: a prospective observational study in Bosnia and Herzegovina, August–December 2020

**DOI:** 10.1016/j.ijid.2021.09.063

**Published:** 2021-11

**Authors:** Sanjin Musa, Esther Kissling, Marta Valenciano, Faris Dizdar, Mia Blažević, Anes Jogunčić, Mirza Palo, Lore Merdrignac, Richard Pebody, Pernille Jorgensen

**Affiliations:** a Institute for Public Health of the Federation of Bosnia and Herzegovina, Sarajevo, Bosnia and Herzegovina; b Sarajevo School of Science and Technology, Hrasnička cesta 3a, Sarajevo, Bosnia and Herzegovina; c Epiconcept, Paris, France; d World Health Organization Office in Bosnia and Herzegovina, Sarajevo, Bosnia and Herzegovina; e Public Health Institute of Canton Sarajevo, Sarajevo, Bosnia and Herzegovina; f World Health Organization Regional Office for Europe, Copenhagen, Denmark

**Keywords:** Household transmission, SARS-CoV-2, COVID-19, Bosnia and Herzegovina

## Abstract

**Background:**

The secondary attack rate (SAR) of severe acute respiratory syndrome coronavirus-2 (SARS-CoV-2) was estimated, and the risk factors for infection among members of households with a coronavirus disease 2019 (COVID-19) index case were identified to inform preventive measures.

**Methods:**

Between 3 August and 19 December 2020, a household transmission study was implemented based on a standardized World Health Organization protocol. Laboratory-confirmed cases of SARS-CoV-2 infection were recruited through the federal COVID-19 database. Trained contact tracers interviewed index cases and household members to collect information on demographic, clinical and behavioural factors. Contacts were followed up for 28 days to identify secondary infections. SAR was estimated and odds ratios (OR) were calculated for risk factors for transmission.

**Results:**

In total, 383 households and 793 contacts were included in this study. The overall SAR was 17% [95% confidence interval (CI) 14–21]. Contacts had higher risk for infection if the primary case had both cough and runny nose (OR 4.31, 95% CI 1.60–11.63), if the contact was aged 18–49 years (OR 4.67, 95% CI 1.83–11.93), if the contact kissed the primary case (OR 3.16, 95% CI 1.19–8.43), or if the contact shared a meal with the primary case (OR 3.10, 95% CI 1.17–8.27).

**Conclusions:**

These results add to the global literature by providing evidence from a middle-income setting. Standard preventive measures in households with positive cases remain critical to reduce transmission.

## Highlights

• The overall severe acute respiratory syndrome coronavirus-2 (SARS-CoV-2) secondary attack rate in this study was 17%.
• Adults were more likely to be secondary cases than children.
• Particular care should be taken if primary cases present with cough and rhinorrhoea.
• Kissing a SARS-CoV-2 case or sharing a meal with a SARS-CoV-2 case increased the risk of infection.
• Reducing contact in the household immediately is key to prevent onward transmission.

## Introduction

In December 2019, severe acute respiratory syndrome coronavirus-2 (SARS-CoV-2), the causative agent of coronavirus disease 2019 (COVID-19), emerged and subsequently spread globally. The World Health Organization (WHO) declared a public health emergency of international concern on 30 January 2020 (World Health Organization, 2020a). As of 16 September 2021, over 224 million laboratory-confirmed cases have been reported worldwide (World Health Organization, 2021). In Bosnia and Herzegovina (population 3.3 million), 224,862 cases were reported between 5 March 2020 and 16 September 2021 (World Health Organization Regional Office for Europe, 2021).

Households are, and will continue to be, important venues for transmission, even in areas where community transmission is reduced. Transmission within households is higher than in other settings (Thompson et al., 2021). For this reason, tracing household contacts for COVID-19 through identification of persons who may have been exposed to COVID-19, and following them up for 14 days from the last point of exposure has been a key response measure (World Health Organization, 2020b).

Household transmission surveys conducted as part of the outbreak response provide a strategic approach to rapidly understand key clinical, epidemiological and virological characteristics of an emerging infection, risk factors for transmission and routes of transmission, and to characterize virus transmission patterns (World Health Organization, 2020c; Bergeri et al, 2021).

Although knowledge of the virological, epidemiological and clinical characteristics of SARS-CoV-2 has progressed considerably since the beginning of the pandemic, many studies have focused on high-income settings. Continued investigation of the transmission of SARS-CoV-2 in medium- and low-income settings, especially as new variants emerge, and understanding the routes of transmission and transmission parameters in a local context remains important to inform and tailor optimal prevention and control measures for COVID-19.

In the Federation of Bosnia and Herzegovina (FBiH), one of the two entities comprising Bosnia and Herzegovina, web-based real-time tracking of suspected COVID-19 cases tested by reverse transcription polymerase chain reaction (RT-PCR) was launched on 27 March 2020. According to official guidelines, the contact tracing programme aims to identify and test all suspected cases of SARS-CoV-2 and their contacts who develop symptoms. Contacts who develop symptoms are advised to get tested by RT-PCR in designated COVID-19 units in outpatient healthcare institutions, outpatient clinics or at drive-through testing locations. Testing may also be done at private laboratories certified by the Federal Ministry of Health. All confirmed and suspected cases are advised to isolate for a minimum of 10 days according to guidelines issued by the Institute of Public Health of FBiH (Institute of Public Health Federation Bosnia and Herzegovina, 2021). Compliance with these measures is overseen by the local health authorities in collaboration with the police, who have the authority to conduct home spot checks and telephone people who have been instructed to self-isolate. Suspected cases that test negative may end self-isolation as soon as the result is available. Using information on cases recorded in this system, the Institute for Public Health of FBiH conducted a study aiming to estimate the secondary attack rate (SAR) of SARS-CoV-2 among household contacts, and identify risk factors for infection in this population to inform optimal preventive measures.

## Methods

### Survey and study design

A prospective study was conducted among households in which one member had tested positive for SARS-CoV-2 by RT-PCR between 3 August and 23 December 2020. Households in nine districts (out of 10) of FBiH were selected, where district public health institutes had engaged contact tracers supported by WHO. All contact tracers had a background in medical sciences and completed a 1-day training course prior to deployment. Contact tracers identified index cases, defined as persons recorded as SARS-CoV-2 positive in the federal real-time web-based database designed to track data on COVID-19 cases. Cases were contacted immediately after laboratory confirmation during the daytime, as required by federal guidelines on contact tracing. Only index cases who reported living with one or more persons when first contacted by the contact tracers were invited to participate in the study, together with all their household members. The recruitment of households took place between 3 and 20 August, and between 15 September 2020 and 27 November 2020. Households were followed-up for a total of 28 days after recruitment. The last follow-up was on 23 December 2020.

A household was defined as two or more persons living in the same apartment or house regardless of kinship. Accommodation facilities such as nursing homes, institutions for the permanent accommodation of persons with special needs, prisons, student accommodation and hostels were not included in this study. A household contact was defined as any person living in the same household as the index case at the time of recruitment. Only persons providing informed consent were included. The study was approved by the Ethics Committee of the Institute for Public Health of FBiH.

### Survey data collection

Contact tracers collected data by telephone using questionnaires from the WHO household transmission investigation protocol for COVID-19 (World Health Organization, 2020c). Index cases and all household contacts were asked about: demographic information (date of birth, gender, occupation, country of residence), household information (household size, number of rooms, number of bedrooms), clinical presentation (including date of symptom onset), healthcare-seeking behaviour (number of visits to health facilities), potential routes of transmission (sharing room, hugging, kissing, sharing a meal, taking care when ill^1^ etc.), laboratory confirmation of SARS-CoV-2 (date of test and date of result), and chronic conditions.

**Figure 1:**
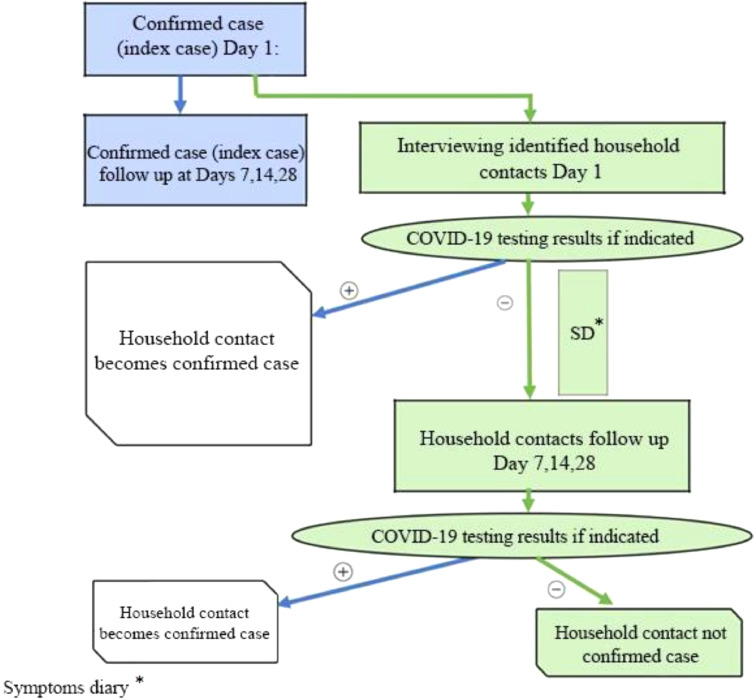
Case investigation algorithm. COVID-19, coronavirus disease 2019.

Contacts were followed-up for 28 days, with interviews on days 1, 7, 14 and 28. Household contacts also completed a daily symptom diary to record the presence or absence of various signs or symptoms. Verbal consent was requested from each participant Fig. 1.

All questionnaires were administered by telephone by the trained contact tracers. Completed forms were sent electronically to the study coordinators at the Institute of Public Health of FBiH and the WHO Country Office in Bosnia and Herzegovina, where data were entered into Go.Data v.2.36.1, a data platform for outbreak investigation and contact tracing (World Health Organization, 2020d).

### Definitions of cases and transmission sequence

Index cases and household members were classified into primary cases, co-primary cases, secondary cases, tertiary cases, non-related cases and non-cases according to the following definitions.

A primary case was defined as the household member with RT-PCR-confirmed SARS-CoV-2 infection or with COVID-19-like symptoms (reporting at least one of cough, sore throat, coryza, shortness of breath, fever, headache or anosmia/ageusia) with the earliest date of symptom onset in the household.

When two or more household members had the same earliest symptom onset date for COVID-19-like clinical manifestations, they were defined as co-primary cases, as was any case with COVID-19-like symptom onset on the day after symptom onset in a primary case. If date of symptom onset was missing, the swab date of a SARS-CoV-2 RT-PCR-confirmed test was taken as a proxy.

A secondary case was defined as a household member with COVID-19-like symptoms within 2–14 days of a primary case, or with an RT-PCR-positive COVID-19 result within 2–14 days of a primary case.

Tertiary cases were defined as household members with COVID-19-like clinical manifestations or laboratory confirmation with symptoms presenting >14 days after a primary case, but within 14 days of a secondary case. Tertiary cases were described but were included as non-cases in the SAR and risk factor analysis.

Household contacts with COVID-19-like symptoms but no onset date, or contacts with other symptoms than COVID-19-like symptoms (e.g. nausea/diarrhoea) and with no positive SARS-CoV-2 RT-PCR result were classified as possible secondary cases. These household contacts were included as non-cases in the main analysis.

Non-related cases were defined as household members with COVID-19-like clinical manifestations or laboratory confirmation presenting >14 days after a primary case, and not within 14 days of a secondary case. These cases were assumed to have been infected outside the household.

All other household members were defined as non-cases.

### Statistical analysis

In the main analysis, households with co-primary cases were excluded, as were households where there was loss to follow-up of contacts within 14 days of the date of symptom onset of the index case (or swab, if onset date was not available).

The explanatory variables were grouped into a conceptual framework to account for their hierarchical relationship. The levels of the framework consisted of household level variables, contact level susceptibility variables (e.g. age of contact, sharing room, sharing meals, etc.) and infectiousness factors related to the primary case (age, sex, symptoms, etc.). Individual multi-variable models were built for each of these three levels to identify risk factors. A combined model was built in a hierarchical way starting with household level variables, contact level variables and, finally, primary case variables. All variables with *P*<0.2 at univariable level were considered for inclusion in the final models. Models were compared using the likelihood ratio test. Variables were retained in the model if the likelihood ratio test *P*-value was <0.1 in the hierarchical steps. Interactions between contact and primary case variables (age and comorbidities) were explored. Sparse data were taken into account when building models.

The serial interval was defined as the time from onset of the first symptom in the primary cases to the time of onset of the first symptom in the secondary case, with a cut-off of 14 days. The same explanatory variables as in the SAR analysis were considered. Survival regression was then undertaken using the best fitting of the log-normal, Gamma or Weibull distributions.

### Sensitivity analyses

In sensitivity analyses, SARs and serial intervals were calculated, including households with co-primary cases. A separate analysis was undertaken considering those households classified as having possible secondary cases as households with secondary cases. All statistical analyses were performed using Stata Release 16 (StataCorp, College Station, TX, USA). Maps were created using ArcGIS Version 27.

## Results

### Descriptive analysis

**Figure 2:**
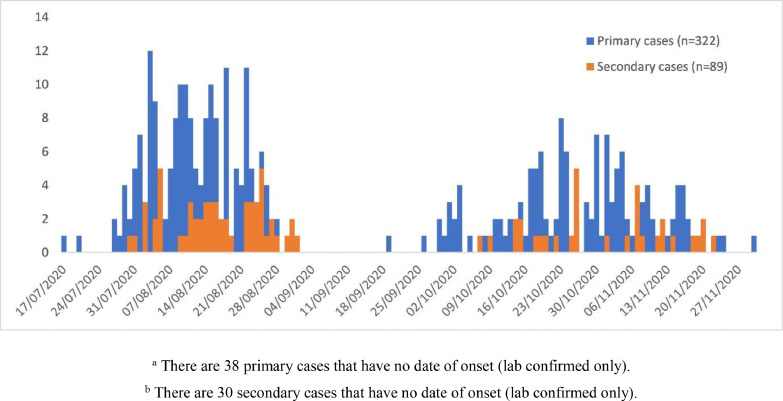
Number of primary cases (*n*=322)and secondary cases (*n*=89)by date of symptom onset of symptoms, Federation of Bosnia and Herzegovina, August 2020–December 2020. ^There were 38 primary cases and 30 secondary cases respectively without date of onset (lab confirmed only)^

Through contact tracing, 1223 index SARS-CoV-2 cases were identified. Of these, 383 (31%) agreed to participate in this study (Figure 2). In total, 793 household contacts were identified, and were classified as 383 primary cases, 21 co-primary cases and 772 household contacts. Among the 103 household contacts (13%) presenting with COVID-19-like symptoms, 58 (56%) were tested by RT-PCR and 43 of them (74%) were positive (Figure S1, see online supplementary material). Among 664 household contacts reporting no COVID-19 symptoms, 143 were tested (22%) and 39 of them (27%) were positive. Five households were lost to follow-up, including five household contacts (Figures S1 and S2, see online supplementary material) and 18 households with at least one co-primary case (Figure S2, see online supplementary material). The main analysis included 360 households, 360 primary cases and 747 household contacts, among whom there were 119 (16%) secondary cases.

**Figure 3:**
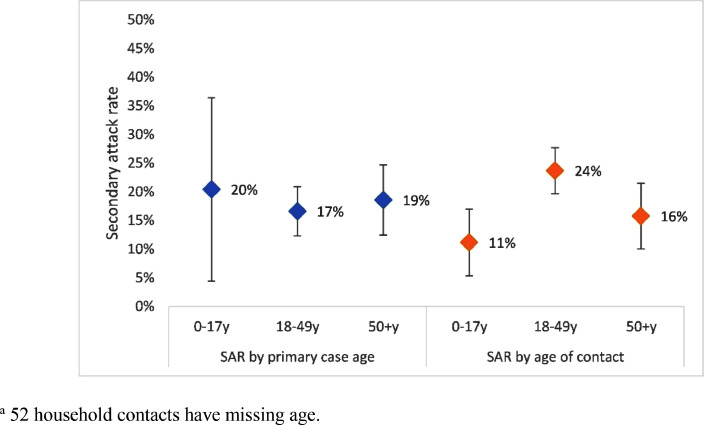
Secondary attack rates by age group of primary cases and household contacts (n=695), Federation of Bosnia and Herzegovina, August 2020–December 2020.Fifty-two household contacts had age missing.

The mean household size was 3.1 persons, with 90% of households containing four or fewer members (Table S1, see online supplementary material). Among primary cases, 89% (322/360) had at least one symptom, with symptom onset between 17 July and 30 November 2020 . Among secondary cases, 75% (89/119) had at least one symptom, with symptom onset between 30 July and 22 November 2020 (Figure 3).

Among primary cases, 52% (187/360) reported fever, 40% (145/360) reported cough, 40% (143/358) reported anosmia/ageusia, 35% (125/360) reported headache and 33% (118/360) reported sore throat (Figure S3, see online supplementary material). Among primary cases, 13% (48/360) did not report any COVID-19-like symptoms. Among secondary cases, 42% (50/119) reported fever, 31% (37/119) reported cough, 21% (25/119) reported anosmia/ageusia, 22% (26/119) reported headache and 23% (27/119) reported sore throat. Symptoms varied by age group (0–17, 18–49 and ≥50 years), with fever being the most common symptom in all age groups (Table S1, see online supplementary material). Among secondary cases, 27% (32/119) did not report COVID-19-like symptoms.

Sixty-three percent of primary cases were aged 18–49 years (228/360). Among household contacts, 58% of secondary cases were aged 18–49 years (65/119), compared with 37% of non-case household contacts (217/682) (Table 1). Among primary cases, 23% (81/360) had at least one chronic condition. Among household contacts, 16% of secondary cases (19/119) had at least one chronic condition, compared with 12% of non-cases (74/682).

**Table 1:** Demographics, medical conditions and outcomes of primary cases of coronavirus disease 2019 and their household contacts, Federation of Bosnia and Herzegovina, August 2020–December 2020.

Characteristic	Values	Primary cases (*n*=360)	Household contacts (*n*=747)	Secondary cases (*n*=119)	Non-cases (*n*=628)^a^
		*n* (%/IQR)	*n* (%/IQR)	*n* (%/IQR)	*n* (%/IQR)
Age (years)	Median age	38 (28–52)	36 (17–54)	34.5 (24–50.5)	37 (16–54)
	Missing	0	52	7	45
Age groups (years)	0–11	4 (1)	113 (16)	13 (12)	100 (17)
	12–17	10 (3)	65 (9)	4 (4)	61 (10)
	18–49	228 (63)	282 (41)	65 (58)	217 (37)
	≥50	118 (33)	235 (34)	30 (27)	205 (35)
	Missing	0	52	7	45
Sex	Male	194 (54)	358 (48)	50 (42)	308 (49)
	Missing	0	0	0	0
Profession: healthcare worker	Yes	36 (10)	19 (3)	4 (3)	15 (2)
	Missing	3	0	0	0
Pregnancy status among women aged 15–49 years	Pregnant	10 (10)	2 (1)	0 (0)	2 (2)
	Missing	12	0	0	0
Medical conditions					
Any chronic condition (not including pregnancy or ‘other chronic condition’)	Yes	81 (23)	93 (12)	19 (16)	74 (12)
	Missing	1	1	0	1
Diabetes	Yes	20 (6)	22 (3)	4 (3)	18 (3)
	Missing	0	1	0	1
Obesity	Yes	28 (8)	25 (3)	9 (8)	16 (3)
	Missing	1	2	0	2
Cancer	Yes	5 (1)	7 (1)	3 (3)	4 (1)
	Missing	0	1	0	1
Heart disease	Yes	20 (6)	37 (5)	10 (8)	27 (4)
	Missing	0	1	0	1
Asthma	Yes	8 (2)	4 (1)	1 (1)	3 (0)
	Missing	0	1	0	1
Chronic lung disease, no asthma	Yes	8 (2)	4 (1)	0 (0)	4 (1)
	Missing	0	1	0	1
Chronic liver disease	Yes	1 (0)	0 (0)	0 (0)	0 (0)
	Missing	0	1	0	1
Chronic haematological disease	Yes	1 (0)	3 (0)	0 (0)	3 (0)
	Missing	0	1	0	1
Chronic kidney disease	Yes	2 (1)	4 (1)	0 (0)	4 (1)
	Missing	0	1	0	1
Chronic neurological disease	Yes	5 (1)	3 (0)	2 (2)	1 (0)
	Missing	0	1	0	1
Organ or bone marrow recipient	Yes	1 (0)	5 (1)	0 (0)	5 (1)
	Missing	0	1	0	1

IQR, interquartile range.

a Includes 11 cases considered as ‘later introduction’ and thus not secondary cases, and two tertiary cases.

In terms of possible routes of transmission to household contacts, 51% (55/119) of secondary cases reported sharing a room with the primary case, compared with 34% (215/628) of non-cases. Among secondary cases, 39% (42/119) hugged the primary case, 34% (36/119) kissed the primary case and 68% (73/119) shared a meal with the primary case, compared with 19% (120/328), 14% (84/628) and 46% (287/628) of non-case household contacts, respectively (Table 2).

**Table 2:** Possible routes of transmission of coronavirus disease 2019 (COVID-19) as reported by household contacts, during illness of the primary case, Federation of Bosnia and Herzegovina, August 2020–December 2020.

Characteristic	Values	Household contacts (*n*=747)	Secondary cases (*n*=119)	Non-cases (*n*=628)^a^
		N (%)	N (%)	N (%)
Shared a room	Yes	270 (37)	55 (51)	215 (34)
	Missing	16	12	4
Took care of case	Yes	121 (17)	30 (28)	91 (15)
	Missing	19	12	7
Hugged case	Yes	162 (22)	42 (39)	120 (19)
	Missing	22	12	10
Kissed case	Yes	120 (17)	36 (34)	84 (14)
	Missing	23	12	11
Shook hands with case	Yes	152 (21)	40 (37)	112 (18)
	Missing	23	12	11
Shared a meal with case	Yes	360 (49)	73 (68)	287 (46)
	Missing	18	12	6
Used same plate as case	Yes	198 (27)	36 (34)	162 (26)
	Missing	18	12	6
Slept in same room as case	Yes	167 (23)	31 (29)	136 (22)
	Missing	15	12	3
Shared toilet with case	Yes	498 (68)	79 (74)	419 (67)
	Missing	15	12	3

### Secondary attack rates

Secondary cases were identified in 89 of 360 households (25%, 95% CI 20–29). The overall SAR was 17% (95% CI 14–21).

SAR decreased with increasing household size (Table 3). In terms of infectiousness factors, SAR was higher in households where the primary case had at least one comorbidity (OR 2.17, 95% CI 0.87–5.41), had a cough (OR 1.77, 95% CI 0.80–3.91), or had a cough and a runny nose (OR 2.31, 95% CI 1.05–5.12). Among susceptibility related factors, SAR was higher among households where the contacts were aged 18–49 years (OR 6.39, 95% CI 2.97–17.23) (Figure 2), shared a room with the primary case (OR 2.94, 95% CI 1.42–6.06), took care of the primary case (OR 4.76, 95% CI 1.99–11.35), hugged the primary case (OR 3.41, 95% CI 1.58–7.33), kissed the primary case (OR 4.16, 95% CI 1.87–9.28), shook hands with the primary case (OR 3.37, 95% CI 1.58–7.19) or shared a meal with the primary case (OR 3.40, 95% CI 11.56–7.41) when the primary case was ill.

**Table 3:** Univariable estimates of observed secondary attack rates (SAR) among households by household level, primary case and household contact characteristics (*n=*747), Federation of Bosnia and Herzegovina, August 2020–December 2020.

Type of characteristic	Characteristic	Value	Secondary cases/all household members^a^	SAR	SAR 95% CI	OR	OR 95% CI
	Unadjusted overall SAR		119/1107	17	14–21		
Household level variables	Household size	2	30/250	23	16–30	Ref	
		3	39/365	16	11–22	0.45	0.17–1.18
		4	33/307	15	9–21	0.39	0.14–1.10
		5+	17/185	12	4–20	0.25	0.07–0.96
	SAR by persons in bedroom^b^	<1 person per bedroom	16/131	21	12–31	Ref	
		1–<2 persons per bedroom	77/653	18	13–22	0.66	0.20–2.16
		2–<3 persons per bedroom	20/229	17	9–24	0.58	0.15–2.29
		≥3 persons per bedroom	6/94	11	1–21	0.27	0.04–1.80
Primary case level variables	SAR by primary case age (years)	0-17	6/50	20	4–36	1.57	0.24–10.17
		18-49	76/742	17	12–21	Ref	
		≥50	37/315	19	12–25	1.27	0.53–3.01
	SAR by primary case gender	Female	54/518	17	12–22	Ref	
		Male	65/589	18	13–23	1.17	0.53–2.58
	SAR by primary case comorbidity status	With no comorbidity	84/869	16	12–20	Ref	
		With at least one comorbidity	35/236	23	15–30	2.17	0.87–5.41
	SAR by healthcare worker status of primary case	No healthcare worker	110/992	18	14–22	Ref	
		Healthcare workers	9/106	15	5–25	0.68	0.17–2.73
	SAR by cough in primary case	No cough	59/657	15	11–20	Ref	
		Cough	60/450	20	15–26	1.77	0.80–3.91
	SAR by cough and runny nose in primary case	No cough or runny nose	43/539	14	9–18	Ref	
		Cough or runny nose	76/568	21	16–25	2.31	1.05–5.12
	Any symptoms in primary case	No symptoms	4/111	7	0–14	Ref	
		Symptoms	115/996	18	15–22	6.28	1.07–36.81
	COVID-19-like symptoms in primary case	No COVID-19-like symptoms	7/134	9	2–17	Ref	
		Covid-19-like symptoms	112/973	18	15–22	3.56	0.86–14.83
Interaction between primary case and contact level variables	SAR by gender interaction	M to M	16/324	14	7–20	Ref	
		M to F	49/265	20	15–25	2.27	0.96–5.38
		F to M	34/228	18	12–23	1.76	0.59–5.26
		F to F	20/290	15	8–22	1.25	0.36–4.28
	SAR by comorbidity interaction	Comorb. to comorb.	9/109	30	15–45	Ref	
		Comorb. to none	26/127	20	13–28	0.37	0.08–1.67
		None to comorb.	10/65	16	8–25	0.23	0.04–1.35
		None to none	74/803	16	12–20	0.21	0.05–0.94
Contact level variables, during illness of primary case	SAR by age of contact (years)	0–17	17/192	11	5–17	Ref	
		18–49	65/510	24	20–28	6.39	2.37–17.23
		≥50	30/353	16	10–21	2.05	0.67–6.25
	SAR by contact's gender	Female	69/555	19	14–23	Ref	
		Male	50/552	16	12–20	0.74	0.42–1.31
	SAR by contact's comorbidity status	No	100/931	17	13–21	Ref	
		Yes	19/174	21	13–28	1.58	0.66–3.76
	Contact shares a room with primary case	No	52/466	12	8–16	Ref	
		Yes	55/278	20	16–25	2.94	1.42–6.06
	Contact took care of primary case	No	77/615	14	10–18	Ref	
		Yes	30/126	26	19–32	4.76	1.99–11.35
	Contact hugged primary case	No	65/571	13	9–16	Ref	
		Yes	42/167	23	17–30	3.41	1.58–7.33
	Contact kissed primary case	No	71/613	13	9–16	Ref	
		Yes	36/124	26	18–34	4.16	1.87–9.28
	Contact shook hands with the primary case	No	67/581	13	9–16	Ref	
		Yes	40/156	24	17–30	3.37	1.58–7.19
	Contact shared a meal with the primary case	No	34/374	10	6–15	Ref	
		Yes	73/368	20	15–25	3.40	1.56–7.41
	Contact used the same plate as the primary case	No	71/541	14	10–18	Ref	
		Yes	36/201	20	14–27	2.12	0.86–5.25
	Contact slept in the same room as the primary case	No	76/573	15	11–19	Ref	
		Yes	31/172	18	13–24	1.56	0.72–3.36
	Contact shared a toilet with the primary case	No	28/237	13	7–18	Ref	
		Yes	79/508	17	13–21	1.85	0.81–4.22

COVID-19, coronavirus disease 2019; OR, odds ratio; CI, confidence interval, M, male; F, female.

a Due to the mixed effects logistic regression with household as random effects, SAR is not equivalent to the quotient of secondary cases divided by household members.

b The number of persons divided by the number of bedrooms – this results in fractions, rather than integers (e.g. 0.75, if three persons and four bedrooms).

Table 4.

Figure 3.

**Figure 4:**
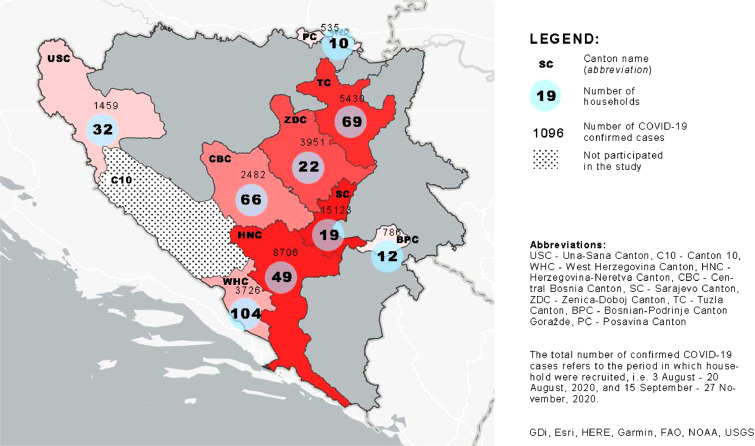
Total confirmed COVID-19 cases and number of households included in the study by cantons of the Federation of Bosnia and Herzegovina.

At multi-variable level, SAR was higher if the primary case had a cough and a runny nose (OR 4.31, 95% CI 1.60–11.63), if the contact was aged 18–49 years (OR 4.67, 95% CI 1.83–11.93), if the contact kissed the primary case (OR 3.16, 95% CI 1.19–8.43), or if the contact shared a meal with the primary case (OR 3.10, 95% CI 1.17–8.27) (Table 4).

### Serial interval

Eighty-four primary–secondary case pairs were used for calculating the serial interval. The best fit for the serial interval model used the Weibull distribution (Akaike information criterion 162.3) (Figure S4, see online supplementary material). The mean serial interval was 6.4 days (95% CI 5.7–7.2). No factors related to household, primary cases (infectiousness factors) or household contacts (susceptibility factors) changed the mean serial interval substantially (Table S2, see online supplementary material).

### Sensitivity analyses

In sensitivity analyses including possible secondary cases, SAR was 18% (95% CI 15–22) and the mean serial interval was 6.7 (95% CI 5.4–8.1). In sensitivity analyses including co-primary cases, SAR was 20% (95% CI 16–23) and the serial interval was 5.6 (95% CI 4.8–6.3).

## Discussion

This study on epidemiological and clinical characteristics of SARS-CoV-2 infections in over 350 households in FBiH during the first year of the COVID-19 pandemic successfully characterized clinical presentation, SAR and potential routes of transmission in an Eastern European setting. SAR was found to be moderate in the household setting. Adults were more likely to be secondary cases than children. Kissing and sharing a meal with the primary case were the main risk factors for infection. Cough and runny nose in the primary case were associated with higher risk of infection.

The overall SAR reported in this study was 17%. This is very similar to pooled estimates presented in recent systematic reviews and meta-analyses of SARS-CoV-2 household studies (Koh et al., 2020; Madewell et al., 2020; Shah et al., 2020), although significant heterogeneity between studies has been observed. Onward transmission from a primary case was recorded in one-quarter of households. This is lower than in some studies (Lewis et al., 2020; Rosenberg et al., 2020; Wu et al., 2020) but similar to other published data (Madewell et al., 2020; Tibebu et al., 2021), and supports evidence of overdispersion in the transmission of SARS-CoV-2 leading to clustering of cases*.* Differences in the proportion of households reporting any secondary transmission from primary cases in this study compared with other investigations could be explained by differences in household size, availability of isolation/quarantine facilities, prevention measures adopted in the families and households, lack of systematic testing of contacts (testing driven by care-seeking behaviour), characteristics of primary cases (e.g. child vs adult) and household composition (e.g. families vs households with students). Finally, this study took place in the second half of 2020 when knowledge about SARS-CoV-2 and non-pharmaceutical interventions had improved considerably, and access to and use of personal protective equipment (e.g. face masks) among the public had increased, which could have contributed to reduced transmission.

**Table 4:** Multi-variable analysis of risk factors for transmission of coronavirus disease 2019 in households (*n*=671^a^), Federation of Bosnia and Herzegovina, August 2020–December 2020.

Type of characteristic	Characteristic	Value	OR	95% CI
Primary case level variable	Cough and runny nose	No	Ref	
		Yes	4.31	1.60–11.63
Contact level variable	Age group of contact (years)	0–17	Ref	
		18–49	4.67	1.83–11.93
		≥50	1.80	0.63–5.20
	Contact kissing the primary case	No	Ref	
		Yes	3.16	1.19–8.43
	Contact sharing a meal with the primary case	No	Ref	
		Yes	3.10	1.17–8.27

OR, odds ratio; CI, confidence interval.

a Excluded cases with missing data: 52 excluded because of missing age, 22 excluded because of missing ‘kissing’ data, and two excluded because of missing ‘sharing meal’ data.

Contacts who were adults had a higher risk of infection from a primary case compared with contacts who were children, in agreement with other studies (mainly in higher income settings) (Madewell et al., 2020). Reduced susceptibility of SARS-CoV-2 infection among children (Galow et al., 2021; Spielberger et al., 2021; Viner et al., 2021a) may explain the lower SAR in this contact group. Nonetheless, asymptomatic and paucisymptomatic infections have been reported to be more common in children than in adults (Viner et al., 2021a). Preferential swabbing of persons with symptoms in this study is likely to have underestimated mild and asymptomatic infections, especially in children, but also the overall SAR as a relatively high proportion of COVID-19 infections are asymptomatic (Yanes-Lane et al., 2020). A higher SAR among younger adults may also reflect greater closeness between spouses, supported by the finding that kissing the primary case was independently associated with SARS-CoV-2 infection in the multi-variable analysis. Finally, younger adults are likely to be main caregivers of both younger and older household members who are ill, increasing their risk of exposure.

Kissing or sharing a meal with the primary case were the only behavioural factors independently associated with increased risk of becoming infected at multi-variable level. Having meals with a SARS-CoV-2-positive case has also been reported as a risk factor for infection in a systematic review (Qiu et al., 2021). In another study, sharing a bedroom was linked to increased transmission, while meal sharing was not (Tek Ng et al., 2021). Several factors associated with increased attack rates at univariable level in this study, including taking care of the case, sharing a room, and hugging, did not remain significant in the final model. As several of these activities are linked, any conclusions derived from multi-variable models regarding the role of specific behaviours should be interpreted with caution. Daily activities in families/households ultimately represent different aspects of very close contact over several days, and it may not be possible to identify the specific activity(ies) that resulted in infection.

Furthermore, persons who had had contact with a primary case presenting with cough and runny nose (rhinorrhoea) had a higher risk of becoming infected compared with contacts of cases without these symptoms. Although presymptomatic and asymptomatic individuals can be infectious, they are less infectious than those presenting with symptoms (Thompson et al., 2021). Coughing, one of the most common COVID-19 symptoms in addition to fever (Grant et al., 2020), has the potential to disperse more virus particles into the surroundings than talking and breathing (Chen et al., 2021). Rhinorrhoea is a less frequently reported symptom of COVID-19 (Lovato and de Filippis, 2020). However, SARS-CoV-2 viral shedding from the nose and nasopharynx has been found to be very high (Gengler et al., 2020), supporting the present findings.

On average, secondary cases presented with symptoms 6.4 days after symptom onset in the primary case. The serial interval falls within the range of estimates from other studies (Alene et al., 2021; Rai et al., 2021). Overall, middle-aged adults presented more frequently with clinical symptoms than children and older adults, except for runny nose (children) and shortness of breath. Children who tested positive for SARS-CoV-2 presented with fewer clinical symptoms than adults, which is similar to observations reported by previous studies (Viner et al., 2021b).

### Strengths and limitations

To the authors’ knowledge, this is the first published household study from a middle-income setting in Europe investigating risk factors for infection, SAR and serial intervals for SARS-CoV-2. The analysis was based on a large prospective study with high completeness, active follow-up of contacts, and a detailed questionnaire.

This investigation had a number of limitations. The analysis may have overestimated SAR as a relatively large proportion of contacts classified as secondary cases had not been confirmed by a laboratory. Also, at the beginning of the study, there were limited public health and social measures in place, including a ceiling on gatherings indoors (maximum of 50 persons) and outdoors (maximum of 100 persons); however, during November 2020, additional measures were implemented, including restrictions on movement and work-from-home policies. Therefore, the possibility that a proportion of secondary cases were infected outside the household cannot be excluded, meaning they were misclassified as ‘household’ secondary cases, hence overestimating SAR. Tertiary cases presenting within 14 days of symptom onset of a primary case may also have been classified erroneously as secondary cases. Conversely, given the presumed high frequency of asymptomatic cases of COVID-19 (Yanes-Lane et al., 2020), the low testing rates of household contacts who did not develop symptoms would lead to under-ascertainment of infections, and thus SAR, especially among children who have asymptomatic infection or present with mild disease more often than adults. Additionally, this study assumed that patients were not secondary cases if their symptoms began <2 days and >14 days after symptom onset in the primary case. Without whole-genome sequencing, temporal sequences of infection cannot be fully validated.

Furthermore, while the study collected information on a large number of variables, data on the relationship between household members, possible community exposure, use of face masks and other personal protective equipment, room ventilation, and previous SARS-CoV-2 infection were not collected. Information on these factors, as well as more detailed data on the exposure variables including the order of magnitude (e.g. number of meals shared, frequency and duration of being in the same room as the index case), would have allowed more precise assessment of the independent risk factors. Data on age were missing for a relatively high proportion of participants, and it was not possible to perform analysis of SAR between different age groups. Information on those factors would have strengthened the findings, as would serological testing of contacts, which was not performed in this study. Finally, although most cantons were included in this study, including rural and urban areas, households were selected by convenience. As such, the results may not be fully representative of the population of FBiH. Moreover, the authors were not able to assess if the moderate participation rate could have resulted in non-response bias.

## Conclusions

This study suggests that measures to reduce contact between confirmed and suspected SARS-CoV-2 cases and other household members, including self-isolation of cases, should be implemented immediately to reduce the risk of onward transmission. Particular care should be taken when primary cases present with symptoms such as cough and rhinorrhoea, and all household members should wear face masks when staying in the same room. Persons who are unable to self-isolate safely at home should be accommodated in other locations when possible. There is a need for evidence-informed campaigns and interventions to increase awareness and compliance with preventive measures within households where one or more members test positive for SARS-CoV-2 in order to reduce transmission in this setting.

Household studies have provided invaluable knowledge on the epidemiology and clinical characteristics of SARS-CoV-2, and continue to be relevant as the pandemic evolves to investigate potential changes in the transmission of emerging variants, including routes of transmission, and potentially as a tool for investigating vaccine effectiveness, and to guide optimal prevention and control measures.

## Disclaimer

The authors alone are responsible for the views presented in this manuscript and they do not necessarily reflect the views, decisions or policies of the institutions with which the authors are affiliated.

## Appendix Supplementary materials

Image, application 1

## Footnotes

1 It should be noted that the questionnaire did not collect data on the frequency and length of these potential exposures.
