# Supplementary material for: Household transmission of SARS-CoV-2: a prospective observational study in Bosnia and Herzegovina, August–December 2020
Source: Int J Infect Dis. 2021 Nov;112:352–61. doi: 10.1016/j.ijid.2021.09.063 (PMC8479489; doi:10.1016/j.ijid.2021.09.063)

# Supplementary information

**Figure S.1**: Flowchart of COVID-19 cases and household contacts, including contacts with Covid-19-like symptoms (as defined by at least one of cough, sore throat, coryza, shortness of breath, fever, headache or anosmia/ageusia), contacts from whom specimens were collected and RT-PCR result, Federation of Bosnia and Herzegovina, August 2020 to December 2020

^a^ 7 persons included here who had onset of symptoms >2 weeks after onset date in the primary case. ^b^ 7 persons (all positive) had specimen date >2 weeks after primary case symptom onset.

Total households

n=383

Primary/co-primary cases
 n=404

Contacts
n=772

Covid-19 symptoms
n=103

Tested
n=58

Pos: 43

Neg: 15

Not tested
n=45

No Covid-19 symptoms
n=664

Tested^b^
n=143

Not tested
n=521

Loss to follow-up:
n=5

Tested
n=1

Not tested
n=4

Pos: 39

Neg: 104

Pos: 1

Neg: 0

**Figure S.2**: Restriction flowchart of the household study, Federation of Bosnia and Herzegovina, Federation of Bosnia and Herzegovina, August 2020 to December 2020

Total households

n=383

Primary/co-primary cases
 n=404

Household contacts
 n=772

Secondary cases
 n=125

Tertiary cases
 n=3

Later intro cases n=11

Not ill
 n=633

Excluding households with loss to follow up: n (household) = 5 households

Primary/co-primary cases
 n=399

Total households

n=378

Household contacts
 n=767

Secondary cases
 n=125

Tertiary cases
 n=3

Later intro cases n=11

Not ill
n=628

Excluding households with co-primary cases: n (households) = 18^a^

Primary cases
 n=360

Total households

n=360

Household contacts
 n=747

Secondary cases
 n=119

Tertiary cases
 n=2

Later intro cases n=11

Not ill
n=615

^a^ Sixteen households with 1 co-primary case, one household with 2 co-primary cases and one household with three co-primary cases.

**Table S.1.** Characteristics of included households, Federation of Bosnia and Herzegovina, Federation of Bosnia and Herzegovina, August 2020 to December 2020

| **Characteristic** | **Values** | N (%/SD) |
| --- | --- | --- |
| Number of households |  | 360 |
| Household size | Mean household size in persons (SD) | 3.1 (1.1) |
|  | Missing | 0 |
| Household size | 2 | 122 (34) |
|  | 3 | 123 (34) |
|  | 4 | 79 (22) |
|  | 5 | 26 (7) |
|  | 6 | 8 (2) |
|  | 7 | 2 (1) |
|  | 8+ | 0 (0) |
|  | Missing | 0 |
| Number of contacts | Mean number of contacts (SD) | 2.1 (1.1) |
|  | Missing | 0 |
| Number of rooms | Mean number of rooms (SD) | 5.8 (2.2) |
|  | Missing | 0 |
| Number of bedrooms | Mean number of bedrooms (SD) | 2.4 (1.1) |
|  | Missing | 0 |
| Number of persons per bedroom | Mean number of persons per bedroom (SD) | 1.5 (0.7) |
|  | Missing | 0 |

**Figure S.3**. Clinical presentation of primary cases and secondary cases among household contacts (n=119), all ages, Federation of Bosnia and Herzegovina, August 2020 to December 2020

**Table S.2.** Clinical presentation of primary cases and household contacts, by age group, Federation of Bosnia and Herzegovina, August 2020 to December 2020

| **Age group** | **Sign / symptom** | **All cases (N=481)** | **Primary (N=360)** | **Secondary cases (N=119)** |
| --- | --- | --- | --- | --- |
|  |  | **N (%)** | **N (%)** | **N (%)** |
| 0-17 years |  | **All cases (N=31)** | **Primary (N=14)** | **Secondary cases (N=17)** |
|  | Fever | 15 (48) | 8 (57) | 7 (41) |
|  | Sore throat | 3 (10) | 2 (14) | 1 (6) |
|  | Cough | 5 (16) | 1 (7) | 4 (24) |
|  | Runny nose | 6 (19) | 2 (14) | 4 (24) |
|  | Shortness of breath | 0 (0) | 0 (0) | 0 (0) |
|  | Chills | 4 (13) | 4 (29) | 0 (0) |
|  | Vomiting | 0 (0) | 0 (0) | 0 (0) |
|  | Nausea | 3 (10) | 3 (21) | 0 (0) |
|  | Diarrhoea | 4 (13) | 3 (21) | 1 (6) |
|  | Headache | 4 (13) | 3 (21) | 1 (6) |
|  | Rash | 0 (0) | 0 (0) | 0 (0) |
|  | Conjunctivitis | 0 (0) | 0 (0) | 0 (0) |
|  | Muscle ache | 0 (0) | 0 (0) | 0 (0) |
|  | Joint ache | 1 (3) | 1 (7) | 0 (0) |
|  | Loss of appetite | 1 (3) | 0 (0) | 1 (6) |
|  | Anosmia/ageusia | 5 (16) | 2 (14) | 3 (18) |
|  | Nose bleed | 0 (0) | 0 (0) | 0 (0) |
|  | Fatigue | 1 (3) | 0 (0) | 1 (6) |
|  | Seizures | 0 (0) | 0 (0) | 0 (0) |
|  | Altered consciousness | 0 (0) | 0 (0) | 0 (0) |
|  | Other neurological symptoms | 0 (0) | 0 (0) | 0 (0) |
| **18-49 years** | **Sign / symptom** | **All cases (N=293)** | **Primary (N=228)** | **Secondary cases (N=65)** |
|  | Fever | 144 (49) | 115 (50) | 29 (45) |
|  | Sore throat | 92 (31) | 72 (32) | 20 (31) |
|  | Cough | 114 (39) | 92 (40) | 22 (34) |
|  | Runny nose | 71 (24) | 59 (26) | 12 (18) |
|  | Shortness of breath | 39 (13) | 32 (14) | 7 (11) |
|  | Chills | 36 (12) | 29 (13) | 7 (11) |
|  | Vomiting | 7 (2) | 7 (3) | 0 (0) |
|  | Nausea | 20 (7) | 19 (8) | 1 (2) |
|  | Diarrhoea | 21 (7) | 20 (9) | 1 (2) |
|  | Headache | 95 (32) | 77 (34) | 18 (28) |
|  | Rash | 2 (1) | 2 (1) | 0 (0) |
|  | Conjunctivitis | 3 (2) | 2 (2) | 1 (3) |
|  | Muscle ache | 72 (25) | 62 (27) | 10 (15) |
|  | Joint ache | 47 (16) | 42 (18) | 5 (8) |
|  | Loss of appetite | 31 (11) | 28 (12) | 3 (5) |
|  | Anosmia/ageusia | 111 (38) | 93 (41) | 18 (28) |
|  | Nose bleed | 3 (1) | 3 (1) | 0 (0) |
|  | Fatigue | 63 (22) | 57 (25) | 6 (9) |
|  | Seizures | 0 (0) | 0 (0) | 0 (0) |
|  | Altered consciousness | 1 (0) | 1 (0) | 0 (0) |
|  | Other neurological symptoms | 0 (0) | 0 (0) | 0 (0) |
| **50+ years** | **Sign / symptom** | **All cases (N=148)** | **Primary (N=118)** | **Secondary cases (N=30)** |
|  | Fever | 73 (49) | 64 (54) | 9 (30) |
|  | Sore throat | 49 (33) | 44 (37) | 5 (17) |
|  | Cough | 60 (41) | 52 (44) | 8 (27) |
|  | Runny nose | 27 (18) | 24 (20) | 3 (10) |
|  | Shortness of breath | 24 (16) | 20 (17) | 4 (13) |
|  | Chills | 24 (16) | 23 (19) | 1 (3) |
|  | Vomiting | 8 (5) | 6 (5) | 2 (7) |
|  | Nausea | 18 (12) | 16 (14) | 2 (7) |
|  | Diarrhoea | 14 (9) | 13 (11) | 1 (3) |
|  | Headache | 50 (34) | 45 (38) | 5 (17) |
|  | Rash | 2 (1) | 2 (2) | 0 (0) |
|  | Conjunctivitis | 1 (1) | 1 (1) | 0 (0) |
|  | Muscle ache | 43 (29) | 41 (35) | 2 (7) |
|  | Joint ache | 23 (16) | 23 (19) | 0 (0) |
|  | Loss of appetite | 41 (28) | 38 (32) | 3 (10) |
|  | Anosmia/ageusia | 51 (34) | 48 (41) | 3 (10) |
|  | Nose bleed | 1 (1) | 1 (1) | 0 (0) |
|  | Fatigue | 45 (30) | 43 (36) | 2 (7) |
|  | Seizures | 0 (0) | 0 (0) | 0 (0) |
|  | Altered consciousness | 1 (1) | 1 (1) | 0 (0) |
|  | Other neurological symptoms | 0 (0) | 0 (0) | 0 (0) |

**Figure S.4.** Unadjusted density of the serial interval in days and fit of log normal, Weibull and Gamma distributions, Federation of Bosnia and Herzegovina, August 2020 to December 2020


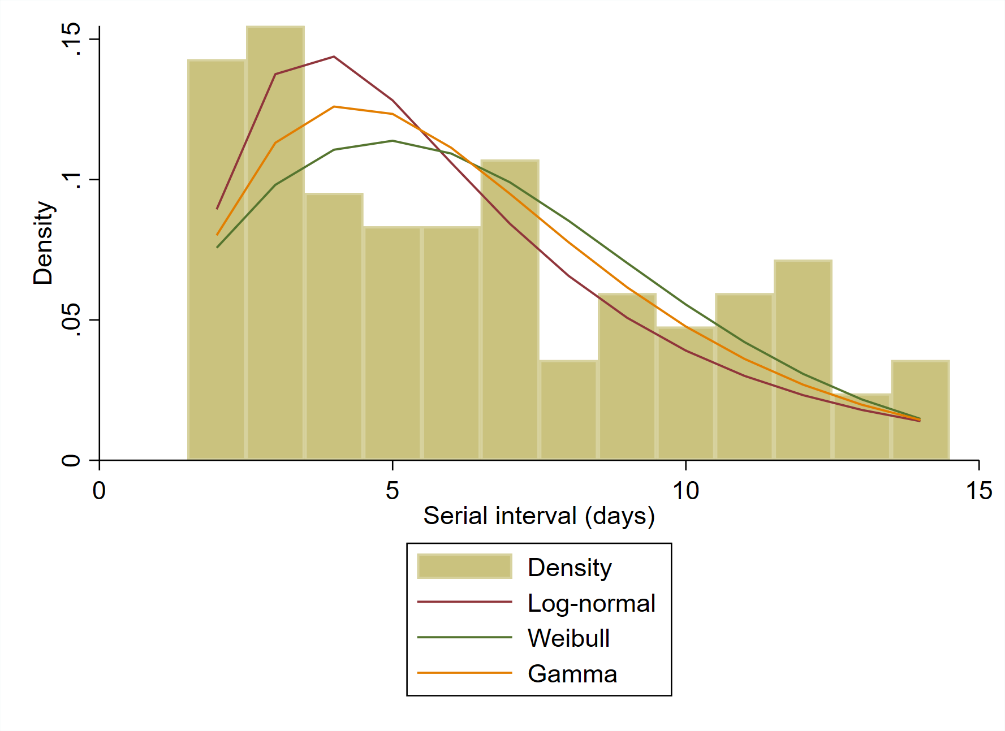

Supplement: Supplementary file 1 [file mmc1.docx]
